# Targeting the tumor microenvironment to enhance antitumor immune responses

**DOI:** 10.18632/oncotarget.3204

**Published:** 2014-12-26

**Authors:** Kevin Van der Jeught, Lukasz Bialkowski, Lidia Daszkiewicz, Katrijn Broos, Cleo Goyvaerts, Dries Renmans, Sandra Van Lint, Carlo Heirman, Kris Thielemans, Karine Breckpot

**Affiliations:** ^1^ Laboratory of Molecular and Cellular Therapy, Department of Immunology-Physiology, Vrije Universiteit Brussel, Laarbeeklaan, Jette, Belgium

**Keywords:** Intratumoral, Immunotherapy, Tumor microenvironment, Immunomodulation, Vaccination

## Abstract

The identification of tumor-specific antigens and the immune responses directed against them has instigated the development of therapies to enhance antitumor immune responses. Most of these cancer immunotherapies are administered systemically rather than directly to tumors. Nonetheless, numerous studies have demonstrated that intratumoral therapy is an attractive approach, both for immunization and immunomodulation purposes. Injection, recruitment and/or activation of antigen-presenting cells in the tumor nest have been extensively studied as strategies to cross-prime immune responses. Moreover, delivery of stimulatory cytokines, blockade of inhibitory cytokines and immune checkpoint blockade have been explored to restore immunological fitness at the tumor site. These tumor-targeted therapies have the potential to induce systemic immunity without the toxicity that is often associated with systemic treatments. We review the most promising intratumoral immunotherapies, how these affect systemic antitumor immunity such that disseminated tumor cells are eliminated, and which approaches have been proven successful in animal models and patients.

## INTRODUCTION

Cancer immunotherapy, instructing the immune system to recognize and kill cancer cells, is a strategy that can result in systemic and selective destruction of tumor cells as well as the formation of memory responses capable of counteracting recurring disease. This concept originated in the beginning of the 20^th^ century when Paul Ehrlich formulated his “immune surveillance hypothesis”. Herein it is postulated that foreign invaders can be recognized and selectively eliminated by the immune system [1]. Over half a century later, Burnet and Thomas, extended this hypothesis to include cancer cells. In their “tumor surveillance hypothesis”, it is proposed that cancer cells are recognized as “foreign” by the immune system [2]. This hypothesis implies that cancer cells can be specifically eliminated without damaging the healthy cells they arise from, in a similar way to virally infected cells.

The identification of antigens that are specifically or preferentially expressed by cancer cells but not by normal tissues offered a rationale for this hypothesis. These antigens are collectively known as tumor associated antigens and are subdivided into several groups according to their expression pattern. The most important classes are cancer-testis antigens, differentiation antigens, viral antigens, mutated antigens and antigens that are over expressed in tumors. Of these, only viral antigens, such as the human papilloma virus E6/E7 proteins and antigens derived from the mutanome are genuinely tumor-specific and foreign to the immune system. All other tumor antigens are considered “self”. Therefore several tolerogenic mechanisms have to be conquered to induce a productive antitumor immune response. Nonetheless, it has been shown that tumor antigens, like cancer-testis antigens, give rise to a number of epitopes. These can be presented in MHC class I or class II molecules to the T-cell receptor of CD8^+^ or CD4^+^ T cells respectively. When this functional recognition coincides with strong stimulatory signals, the CD8^+^ and CD4^+^ T cells are driven towards cytotoxic T lymphocytes (CTLs) and T helper type 1 (T_H_1) cells respectively. Attempts to stimulate immunity against tumor antigens and consequently against the entire tumor have been undertaken based on the extensive research on the immunogenicity of these antigens [3].

**Figure 1:**
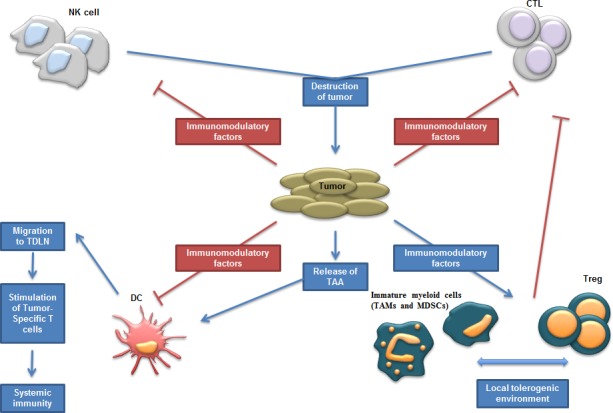
Schematic representation of tumor environment; interactions between tumor and immune cells Different immune cell types mediate the destruction of tumor cells such as NK and T cells. In contrast to the direct effects exerted by NK cells, adaptive T-cell responses are generated once DCs have primed T cells in the draining lymph nodes. However, the tumor modifies the environment in order to disrupt these antitumor effects and recruits and stimulates suppressive immune cells. Abbreviations: CTL (Cytotoxic T Lymphocytes); DC (Dendritic Cell); MDSC (Myeloid-Derived Suppressor Cell); NK cell (Natural Killer cell); TAA (Tumor-Associated Antigen); TAM (Tumor-Associaded Macrophages); TDLN (Tumor-Draining Lymph Nodes); Treg (Regulatory T cells).

Most cancer immunotherapies are administered systemically rather than directly into tumors, despite the pioneering work of William B. Coley, who demonstrated that heat-inactivated bacterial cultures from *Streptococci* and *Serratia marcescens* could induce tumor regression, and that the therapy outcome was most favorable when these bacteria were injected in the proximity of the tumor [4]. The growing knowledge on tumor immunology provides a rationale for Coley's findings. In response to tumor-mediated factors, infiltrating immune cells like dendritic cells (DCs), natural killer (NK) cells and CTLs are suppressed, whilst other cell types, such as myeloid-derived suppressor cells (MDSCs), tumor-associated macrophages (TAMs) and regulatory T cells (Tregs), are exploited to the tumor's advantage (Fig. 1). Importantly, tumor-resident antigen-presenting cells, such as DCs that carry tumor antigens, can become activated by pathogen-associated molecular patterns (PAMPs). Consequently, they escape the immunosuppressive tumor environment and migrate to the tumor-draining lymph nodes where they can prime T cells. Despite the fact that cross-priming of tumor-specific T cells is a critical step in anticancer immunotherapy, it does not automatically imply tumor regression. This is explained by the presence of numerous T-cell suppressive factors within the tumor nest. Therefore, therapies that modulate key suppressive factors are equally important as strategies that drive T-cell stimulation. However, the immune system has still its mysteries, as for instance immunosuppressive drugs such as rapamycin and rapalogs (rapamycin-like compounds) can be used for cancer prevention and therapy [5-7]. Therefore, careful analysis of which compounds will be manipulated in the immune system need to be taken into account.

This review provides a comprehensive summary of the state-of-the-art on intratumoral immunotherapies, including both stimulation and immunomodulation approaches.

## INTRATUMORAL IMMUNIZATION

### Intratumoral injection of dendritic cells

The era of DC vaccination was unleashed by the pioneering work of Inaba *et al.* [8], who showed that mouse DCs could be cultured *ex vivo* from bone marrow cells. In 2011, Ralph Steinman received the Nobel Prize for the discovery of this cell type and its role in adaptive immunity. The primary objective of DC-based antitumor vaccination is to achieve stimulation of long-lived cancer-specific T-cell responses. For this purpose, *ex vivo* generated DCs are typically loaded with tumor antigens and simultaneously or subsequently matured [9].

Much debate exists on the optimal way to generate and mature DCs *ex vivo* and on the possible clinical failure due to suboptimal DC maturation. A detailed description on the generation of DC vaccines is provided in [9, 10]. Another important matter concerns the choice of antigens and the delivery platform. The latter has been examined in detail elsewhere [11, 12]. Even supposing that an ideal mix of antigens is found, a recurrent issue is the simultaneous evolvement of tumors and their antigens. The loss of antigens is a mechanism that is exploited by tumor cells to escape antitumor immune responses [13]. Therefore, loading of DCs with “classical” tumor antigens might in part explain why only a temporary control of tumors is observed in many experimental settings.

To circumvent these issues, delivery of “empty” DCs to the tumor has been explored. This idea is based on the presumption that the DCs will capture antigens released by the tumor and drain to lymphoid organs to present their cargo to T cells. This concept was evaluated by Hirao *et al.* [14], who showed that *ex vivo* generated mouse DCs can migrate from the tumor to draining lymph nodes and cross-prime tumor-specific T_H_1-responses. However, analysis of the migration of intratumorally injected human DCs showed that most of them remain inside the tumor. It was found that tumors from hepatocellular carcinoma, colorectal or pancreatic cancer patients produced IL-8; a chemokine that attracts DCs and that blocks their migration towards MIP-3β (CCL19), a cytokine that draws DCs to lymphoid organs [15]. Of note, mice do not express a homologue for human IL-8, therefore the retention of DCs due to IL-8 production cannot be investigated in mice [16]. Nonetheless, the approach of delivering DCs into the tumor has been further explored.

Since activation of T_H_1 cells and CTLs requires strong co-stimulation, the “next generation intratumoral DC-based vaccines” were composed of DCs modified to express high levels of T-cell promoting cytokines either or not combined with co-stimulatory molecules [17]. An example of such experimental setting is the use of DCs modified to express IL-12; a heterodimeric cytokine with various immune promoting functions. Nishioka *et al.* [18] demonstrated that bone marrow-derived DCs that were retrovirally transduced to express high levels of IL-12, were able to evoke antitumor immune responses capable of mediating regression of established tumors, including the weakly immunogenic B16 melanoma. This result could not be obtained when delivering non-transduced DCs or IL-12 producing fibroblasts, indicating the need for both IL-12 and potent antigen-presenting cells. Satoh *et al.* [19] confirmed these findings showing therapeutic antitumor immune responses in a model of colorectal cancer upon delivery of DCs that were modified with adenoviral vectors harboring the IL-12 gene. However, it must be acknowledged that although the effects were attributed to the presence of IL-12, the viral vectors used in these studies have the ability to induce DC maturation [20, 21]. Since it was shown in mice with metastatic prostate cancer that intratumoral immunization with DCs over-expressing both IL-12 and the co-stimulatory molecule B7.1 (CD80) resulted in less lung metastases than immunization with DCs expressing only IL-12 [22], it cannot be excluded that other maturation-inducing factors contributed to the outcome observed in the Nishioka [18] and Satoh [19] study.

To further improve immunization upon intratumoral delivery of DCs, several research groups have combined the expression of IL-12 with other cytokines, including IL-18 [23], or IL-21 and IFN-α [24]. These studies showed that DCs modified to produce multiple stimulatory cytokines were more efficient in stimulating therapeutic T-cell responses than DCs producing only IL-12.

The use of monocyte-derived DCs modified with adenoviral vectors harboring the IL-12 gene was translated to the clinic by the group of Ignacio Melero [17]. Seventeen patients were treated in a pilot study; 3, 5 and 9 of whom suffered from metastatic pancreatic, colorectal or primary liver malignancies respectively. These patients received 3 intratumoral injections of DCs at escalating doses at a 21-day interval. The most common side effects were lymphopenia, fever and malaise. Antibodies against adenoviral components were also detected. As these hamper repeated injection of the vaccine, they are also considered as adverse events. Importantly, immune reactions were observed in these patients: elevated IFN-α and IL-6 serum levels were detected in 15 patients, increase in NK cell activity was observed in 5 patients and 3 patients showed presence of tumor-infiltrating CD8^+^ T cells. Moreover, a partial response was observed in 1 patient with pancreatic carcinoma, whereas stabilization of the disease was observed in 2 patients. This study showed that delivery of IL-12 producing DCs to the tumor is feasible and well tolerated but further improvements are required to increase clinical efficacy.

Taken together, the pre-clinical and clinical studies described above suggest that DCs delivered to the tumor are able to ingest tumor antigens, which can then be presented to effector immune cells. Moreover, modifications of DCs to secrete cytokines can further enhance the efficacy of this approach.

### Intratumoral injection of cytokines to recruit dendritic cells

The direct injection of *ex vivo* generated DCs is a straightforward strategy to ensure an increase of DC numbers in the tumor environment. However, the generation of DCs *ex vivo* is costly, laborious and time-consuming. In addition, it is known that DCs are a heterogeneous population of antigen-presenting cells; several DC-subsets, endowed with distinct functions, have been described in mice and men [25]. It is currently unclear, which DC-subset is most suitable for vaccination. In addition, it remains a major challenge to generate high numbers of DCs that resemble a certain subset of *in vivo* existing cells [26]. Therefore, recruiting DCs directly *in vivo* offers an attractive alternative.

Several strategies have been employed to attract DCs to the tumor. They typically depend on delivery of growth factors, such as Flt3L and GM-CSF [27-29]. Both factors have been intensively studied for the *in vitro* generation of DCs, and data showed that Flt3L-derived DCs share morphological characteristics and surface antigens with normal lymphoid-derived DC-subsets activated with LPS or IFN-α [30]. In contrast, DCs cultured in GM-CSF express myeloid cell surface antigens such as CD11c and lack the expression of CD8α; therefore they are considered to be the progeny of cells of the myeloid lineage [8]. *In vivo* Flt3/Flt3L-mediated signaling regulates the generation and differentiation of plasmacytoid, resident and migratory DCs from bone marrow progenitor cells [31, 32]. In earlier days subcutaneous injection of Flt3L in tumor bearing mice has been used as a strategy to increase DC numbers and mediate tumor control [33, 34]. However, the intratumoral delivery of Flt3L has shown variable outcome. It was shown in a hepatoma, colorectal cancer (SMCC-1) and breast cancer model that adenoviral delivery of Flt3L to the tumor resulted in an increase in DCs and tumor-specific T cells, and as such delayed tumor growth [35, 36]. Combining the delivery of Flt3L with the induction of tumor cell apoptosis enhanced the therapeutic effect. In contrast, Riediger *et al.* [37] failed to show the therapeutic potential of adenoviral delivery of Flt3L in CT26 colorectal tumors. The latter might be due to differences in immunogenicity between the SMCC-1 and CT26 colorectal tumor models. Importantly, this highlights the risk of extrapolating data from one model to the other.

The application of GM-CSF as a cytokine to attract immature DCs to the tumor was first demonstrated by Pan *et al.* [38]. Using a model of hepatic metastatic colon cancer they showed that intratumoral delivery of GM-CSF resulted in the recruitment of DCs as well as the capture of antigens and subsequent maturation of these DCs. This in turn enabled them to migrate to lymphoid tissues, where they were able to activate antigen-specific T cells. Moreover, intratumoral delivery of plasmid DNA encoding GM-CSF resulted in an increased number of immature DCs [39]. However, this was not enough to induce effective antitumor activity; it was hypothesized that GM-CSF lead to expansion of the already infiltrated (and functionally impaired) DCs. Only co-delivery of CCL20 (MIP-3α) with GM-CSF resulted in tumor antigen (MUC1)-specific antitumor immune responses. Therefore, it was suggested that CCL20 was responsible for the recruitment of novel functional DCs to the tumor and that GM-CSF triggered the subsequent expansion and maturation of the recruited cells [40, 41].

It is known that DCs within the tumor environment are under the influence of a plethora of inhibitory mechanisms, which impairs their maturation process. It has been proposed that strong adjuvants can jumpstart the activation program in DCs. In this regard, Davis *et al.* [42] have combined the use of GM-CSF with the delivery of LPS, a strong activator of the pattern recognition receptor (PRR) TLR4, achieving an increase in the number of activated antigen-presenting cells in the tumor environment. They showed in several tumor models, including the melanoma model B16 and the colorectal model CT26, that this treatment strategy leads to an overall decrease in tumor volume.

GM-CSF has also been delivered by oncolytic viruses (Herpes simplex virus-1, HSV-1) to lesions of patients with metastatic melanoma [43, 44]. This strategy was well tolerated and melanoma lesions treated with these viruses exhibited a higher number of MART-1-specific T cells, whilst showed a reduction in Tregs and MDSCs. More importantly, a 26% objective response rate and 1-year survival rate of 58% was observed for all patients. The duration of the response was between 16 and 40 months. It is hypothesized that this approach takes advantage of the induction of tumor cell death and attraction of DCs. Tumor cell death generates phagocytic material for antigen-presenting cells [45] and provides damage-associated molecular patterns (DAMPs) that are able to activate DCs. These DAMPs include uric acid, heat shock proteins, adenosine triphosphate and high mobility group box 1 proteins [46-49]. Immune activation upon induction of virus-mediated tumor cell death was even reported in the absence of these DAMPs, suggesting that viral components might suffice for an activation of the tumor-infiltrating DCs [14, 50, 51].

In conclusion, these studies reveal that DCs might be attracted to the tumor using different growth factors. In addition, they suggest that DC recruitment is a first step that needs to be followed by antigen loading and subsequent DC activation to induce potent antitumor immune responses.

### Intratumoral delivery of dendritic cell potentiating factors

Professional antigen-presenting cells actively infiltrate tumor lesions. However, these cells are subjected to immunosuppressive mechanisms, which inhibit them to perform properly. Two main DC potentiating strategies will be reviewed in this section: triggering of TLRs and CD40. Additionally, we will discuss a number of co-stimulatory molecules that have been used to enhance T-cell mediated responses.

#### Intratumoral delivery of TLR agonists

TLRs are members of the PRR family of which the genetic sequence is widely conserved over distant organisms. Until now, 13 TLRs have been characterized in mammals, of which 10 are also found in humans [52]. A broad range of PAMPs and DAMPs is recognized by different TLRs. Notably, the function of TLRs is not restricted to innate immune responses as initially thought; TLRs also play a role in tissue repair and in adaptive DC and T cell-mediated immune responses against cancer [53]. Interestingly, the very first immunotherapy approaches applied by Busch, Coley and others were in fact taking advantage of the activation of the immune system through these TLRs [52, 54].

Growing evidence suggest that the delivery of TLR-agonists into the tumor nest (locally) is preferred over its systemic administration. Besides an improved outcome, this strategy decreases the side effects related to systemic TLR-agonist administration, such as the induction of uncontrolled and overwhelming inflammation [55].

An important issue when using TLR-agonists for intratumoral delivery is related to the choice of the best-suited agonist for DC activation and thus for the induction of antitumor immunity. Of note, distinct DC subsets are shown to preferentially express certain TLRs [56, 57]. This differential TLR expression pattern reflects the functional specialization of DC populations, which depending on the nature of an invader can direct the appropriate immune response.

Amongst all TLRs, TLR4 is the best characterized. TLR4 is strongly activated by LPS; its systemic administration leads to a massive release of pro-inflammatory cytokines and as a result so-called endotoxic shock. However, combining DC mobilization with TLR4-triggering was efficacious for the treatment of different murine tumor models [42, 58]. Therapeutic potential of intratumoral administration of immature DCs with topical application of a TLR7 agonist (Imiquimod) was also demonstrated in a mouse melanoma model [59]. The effect of Imiquimod seems to depend on local administration, since its systemic delivery was previously shown to be ineffective for the treatment of human carcinomas [60]. The use of TLR7 agonists without additional delivery of DCs to the tumor was also investigated in a murine breast cancer model, showing that TLR7-triggering on infiltrating plasmacytoid DCs resulted in their activation. This activation lead to high expression of type I IFNs, which contributed to the therapeutic effect observed in this model [61].

The immune activating capacity of CpG-ODN, a TLR9 agonist, upon intratumoral delivery was illustrated by Shirota *et al.* [62], who showed that monocytic MDSCs lost their suppressive activity upon TLR9 triggering and acquired a tumoricidal phenotype. Of note, this group previously reported that these effects were not obtained upon systemic administration of CpG-ODN [62]. Remarkably, several phase I clinical trials in which CpG-ODN was delivered to the tumor to treat malignant melanoma yielded promising results. Hofmann *et al.* [63] achieved tumor regression in half of the treated subjects using this strategy, whereas Molenkamp *et al.* [64] and Brody *et al.* [65] showed that CpG-ODN administration, either alone or combined with radiotherapy induced tumor regression, accompanied by the generation of tumor-specific CD8^+^ T cells.

Two recent studies report on the effect of intratumoral administration of CpG-ODN and polyI:C, a potent TLR3 ligand, and their ability to convert the chronic inflammatory tumor environment into an acute inflammatory environment. This was shown to rescue the functionality of CD8^+^ T cells that were adoptively transferred or generated through vaccination [66, 67]. Increased efficacy of the applied immunotherapeutic approach in these studies was linked to the activation of tumor-infiltrating DCs, as characterized by the enhanced expression of co-stimulatory molecules and pro-inflammatory cytokines. These data show that the administration of TLR agonists such as polyI:C, LPS, imiquimod and CpG-ODN, has therapeutic anticancer potential. However, it needs to be clarified which agonist has the best curative profile. Only a few studies addressed this question; Shirota *et al.* [62] evaluated whether triggering of TLR2, 3, 4 and 8 on MDSCs had a similar effect as described for the stimulation of TLR7 and TLR9, including the differentiation of the MDSCs to a tumoricidal, macrophage-resembling phenotype. Although MDSCs express TLR2, 3, 4 and 8, triggering of these TLRs did not result in MDSC-differentiation. Intratumoral delivery of CpG-ODN in the murine glioma model GL261 inhibited tumor growth *in vivo* and cured 80% of the mice [68]. Subsequently, they demonstrated increased frequencies of tumor-infiltrating IFN-α producing effector T cells and a marked increase in the ratio of CD4^+^ effector T cells to CD4^+^ Foxp3^+^ Tregs. They evidenced in this study that only intratumoral injection of CpG-ODN, Pam3Cys-SK4 or R848 stimulating TLR9, 1/2 and 7/8 respectively, leads to survival benefit, whereas stimulation of TLR3 or 4 by injection of poly(I:C) or LPS alone is not effective. This comparative study highlights that TLR-agonists that might be successful in one model might not necessarily be effective in another [68]. This might have several explanations; the work of Shirota et al. [62] on MDSCs and their phenotype after stimulation with various TLR-ligands, suggests that the composition of immune infiltrate might play an important role. It is also known that tumor cells themselves express various TLRs and their stimulation leads to a different effect than triggering of the same TLRs on immune cells. For instance, it has been shown that intratumoral administration of LPS can induce resistance to CTLs and drives NK cell anergy [69], whereas triggering of TLR3 on certain tumor cell types results in tumor growth arrest [70]. Therefore, triggering of TLRs can be considered a double edged sword, as it can also be protumoral [71]. This is highlighted by the fact that in some cases triggering of TLRs on cancerous cells can result in promotion of tumor growth, evasion and chemoresistance [69, 72-74]. Additionally, signaling upon TLR-activation is tightly regulated; this control includes several negative activation loops, which have been reviewed in detail elsewhere [75, 76]. The presence of soluble decoy receptors like soluble TLR2 and TLR4 and the down-regulation of TLR expression by anti-inflammatory cytokines, such as TGF-β and IL-10 are the most likely functional mechanisms in the tumor environment. Delivery of constitutively active TLRs (caTLRs) to immune cells using viral or non-viral methods allows evaluation of their performance on immune cells, whilst circumventing the inhibitory mechanisms. These caTLRs and their use for DC-activation, have been reviewed by Breckpot *et al*. [53]. Our group observed that intratumoral administration of mRNA encoding caTLR4 leads to the induction of antigen-specific CTL responses but this is not correlated with a therapeutic benefit (unpublished data). This observation points out the necessity of adding additional factors to achieve strong antitumor immune responses.

#### Intratumoral delivery of CD40L

The CD40/CD40L interaction was shown to be crucial for the induction of potent CTLs [77]. CD40L, being expressed on activated CD4^+^ T cells, was found to license the antigen-presenting cells endowing them with stronger antigen presentation capacities as well as reinforcing the expression of co-stimulatory molecules on their surface [78]. Blockade of CD40L decreases the number of antigen-specific CTLs, as shown by the group of Cornelis J. M. Melief [78]. Based on these findings, new therapies were proposed to activate the CD40/CD40L pathway.

Several tumor models with various histological origin were transduced *in vivo* by means of intratumoral administration of an adenoviral vector encoding CD40L [79-85]. These studies gave satisfying results in most of the tested models: intratumoral CD40L-expression led to strong antitumor responses and caused tumor regression in a dose-dependent manner. *In vivo* depletion of CD4^+^ and/or CD8^+^ T cells confirmed involvement of both cell types in the generated immune response, although CD8^+^ T cells came out as the major effectors. Moreover, the treatment induced T-cell memory, as tumor-free animals were protected against re-challenge. The role of CD40/CD40L stimulation in the induction of anticancer immune responses was confirmed using agonistic anti-CD40 antibodies [85]. It was also demonstrated that intratumoral administration of anti-CD40 antibodies was effective in licensing strong systemic CTL immunity, resulting in eradication of distant tumor nodules without side effects. Noteworthy here is the use of a CD40^−^ tumor model, which confirms the hypothesis that DCs are the most likely targets for the agonistic anti-CD40 antibodies [85]. Importantly, distinct mechanisms of action of anti-CD40 antibodies are described in the murine and human setting. Recent studies in mice showed the requirement for cross-linking of the Fc-receptor to generate potent antigen-presenting cell activation [86]. The clinical effects of the anti-human CD40 mAb, CP-870.893, are however independent of Fc-receptor cross-linking [86-89]. Meanwhile, it was shown that expression of CD40L on tumor cells facilitates their interaction with DCs leading to DC maturation, secretion of cytokines and to formation of T-cell dependent antitumor immunity [90]. Besides stimulation of CTL responses, agonistic anti-CD40 antibodies are known for targeting CD40^+^ tumor blood vessels, hereby enhancing tumor neoangiogenesis, which could give a pro-tumorigenic result [91, 92]. Whether the outcome of anti-CD40 antibody therapy is pro- or antitumorigenic will most likely depend on the immunogenicity of the tumor. Therefore, it is of interest to develop strategies that allow triggering of the CD40 pathway on antigen-presenting cells, whilst minimize the activation of endothelial cells in the tumoral blood vessels. The group of Ronald Levy investigated the feasibility of combining CD40L therapy with chemotherapy in the treatment of murine lymphoma [84]. They showed that this combination resulted in a complete tumor regression and a long-term survival of mice. Importantly, in this combinatorial treatment, the observed effect was due to the direct intratumoral injection of the CD40L virus, as peritumoral delivery or injection at distant sites did not result in a same therapeutic outcome.

#### Combining TLR agonists with co-stimulatory signals

Based on the promising results obtained with TLR- and CD40-triggering, Ahonen *et al.* [93] showed for the first time that the combination of different TLR agonists synergized with CD40/CD40L pathway. This stimulation resulted in the induction of potent CD8^+^ T-cell expansion that was dependent on type I IFN. B16F10 tumor-bearing mice were intratumorally injected with a plasmid containing soluble CD40L together with different TLR agonists [94]. The CD40-activation by itself already slowed tumor growth and prolonged survival. Combined with agonists of TLR3 and TLR9 (polyI:C and CpG respectively), intratumoral CD40L-administration greatly enhanced this therapeutic effect. This was found to be associated with a reduction in intratumoral CD11c^+^ cells and an increase in CD8^+^ T-cell number.

In addition to the combination of TLR and CD40/CD40L triggering, different co-stimulatory molecules were tested to improve T-cell mediated responses. Other TNF superfamily members including 4-1BBL, OX-40L, CD70 and GITRL were evaluated for their possible use in antitumor therapies. These family members were reviewed elsewhere [95-99].

To illustrate the potential of co-stimulatory molecules, Murphy *et al.* [100] investigated the use of different fusion proteins of co-stimulatory molecules (Fc-GITRL, Fc-OX40L, Fc-4-1BBL and CD80-Fc) in a murine glioma model. A tumor lysate vaccine was injected intradermally to induce primary immune responses and followed by systemic administration of different co-stimulatory molecules. Solely the combination of Fc-OX40L with the vaccine led to 50 % long-term survivors, while Fc-GITRL led only to a delayed tumor growth. No effects were observed when CD80-Fc and Fc-4-1BBL were used. Analysis of T-cell responses after administration of the different fusion proteins showed a correlation between the survival of the animals and the presence of tumor-resident and tumor-draining lymph node-resident lymphocytes. Mice treated with the vaccine/Fc-OX40L fusion showed an increase in CD4^+^ and CD8^+^ T-cell proliferation. Re-challenge of the long-term survivors led to tumor rejection, indicating that immunologic memory against the tumor cells was established [100].

The use of molecules that block inhibitory signals, trigger co-stimulatory signals in combination with the stimulation of TLRs was shown by Marabelle *et al.* [101] who injected a TLR9 agonist (CpG nucleotides) together with anti-CTLA-4 or anti-OX40 antibodies intratumorally to deplete tumor-resident Tregs. This low-dose *in situ* immunotherapy resulted in systemic immune responses leading to the eradication of established tumors at distant sites and a prolonged survival [101].

**Figure 2:**
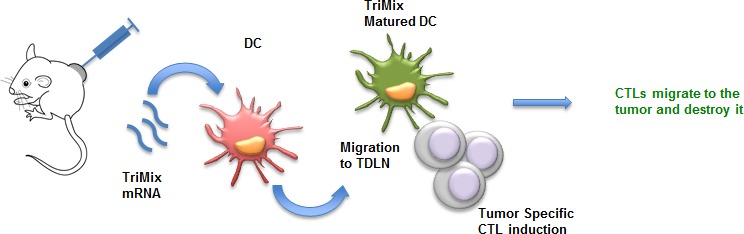
mRNA encoding for TriMix as a tool for intratumoral immunization Upon intratumoral delivery, mRNA encoding TriMix (CD40L, caTLR4 and CD70) is taken up by DCs. These tumor-residing DCs pick up tumor antigens, mature upon translation of the TriMix mRNA and migrate towards the tumor-draining lymph nodes. Fully matured DCs induce tumor-specific CTLs that in turn migrate back to the tumor. Abbreviations: CTL (Cytotoxic T Lymphocytes); DC (Dendritic Cell); TDLN (Tumor-Draining Lymph Nodes).

In 2008 our group developed a DC vaccination platform in which autologous DCs are electroporated with a mixture of mRNA molecules encoding for CD40L, caTLR4 and CD70, collectively called TriMix [102]. This technology was successfully applied in a phase Ia clinical trial to treat advanced melanoma patients [103]. However, as the tumor-associated antigens are not always known/available, we aimed to test the direct intratumoral injection of TriMix mRNA (Fig. 2). Upon this treatment, tumor-resident DCs were shown to specifically engulf and express the mRNA and were reprogrammed to mature and migrate towards the tumor-draining lymph nodes [104]. This resulted in the induction of tumor-specific CTLs in different tumor models. Most importantly, we were able to induce an immune response against a neo-epitope presented by the P815 mastocytoma tumor model. Therapeutic experiments showed systemic antitumor responses; providing a rationale to apply this approach in a clinical setting.

## INTRATUMORAL IMMUNOMODULATION

### Delivering immunomodulating cytokines

Intratumoral delivery of cytokines, either as proteins or encoded by genes, is a strategy that has entered the clinic over a decade ago [105]. This approach is characterized by three main requirements: (i) a high concentration of the immunomodulatory cytokine inside the injected tumors; (ii) the persistence of the cytokine at a sufficient concentration to trigger a therapeutic response and finally (iii) a low, non-toxic concentration of the cytokine outside the tumor nest [106]. Several cytokines including IL-2, IL-12, TNF-α, type I IFNs and GM-CSF possess anticancer potential. Since GM-CSF was discussed above, we will focus in this section on the other cytokines.

### Intratumoral delivery of IL-2

IL-2 is a glycoprotein that is best known as a growth factor for T and NK cells. Upon binding to its receptor, IL-2 activates multiple signaling pathways, which promote cell survival, proliferation and cytolytic functions [107]. Notably, IL-2 also supports the survival and proliferation of Tregs [108] and upon prolonged administration to T cells it can induce activation-induced cell death [109]. Nonetheless, systemic administration of IL-2 was shown to exert anticancer potential. This activity, mediated by CD8^+^ T cells, has led to its approval as an anticancer agent by the FDA in 1998 after a meta-analysis of 270 patients in a total of 8 clinical trials. However, IL-2 systemic administration is also associated with adverse events, such as pulmonary edema, low blood pressure and low systemic vascular resistance as well as hematologic, hepatic and renal toxicity [110]. Although these side effects are often reversible, it is advisable to deliver IL-2 locally. Gutwald *et al.* [111] were the first to report on the peritumoral injection of IL-2 in metastasized melanoma. In contrast to its systemic delivery, intratumoral administration of IL-2 was well tolerated in patients [112]. In the study of Weide *et al.* [113], 72 patients were treated with 3 intratumoral injections of IL-2 weekly until clinical regression was achieved. This trial demonstrated the long-term benefit of intratumoral delivery of IL-2 in stage III patients without lymph node involvement and in stage IV patients with soft tissue metastasis but without visceral involvement. These patients maintained therapeutic responses even when the treatment was stopped. Optimized delivery techniques could enhance these favorable clinical effects. Zhao *et al.* [114] showed in a preclinical model with dextran PLGA-PLA microspheres, which continuously releases IL-2, the benefit of a single intratumoral IL-2 administration, as compared to multiple injections. This highlights the importance of sustained local high levels of cytokines to achieve therapeutic and immunological responses. Alternative strategies have been developed to deliver IL-2; including adenoviral vectors encoding IL-2 and delivery using *in vivo* electroporation [115]. These strategies have shown clinical safety and feasibility but without a clear therapeutic benefit for patients [115, 116]. In another attempt to circumvent systemic toxicity, IL-2 was fused to carcinoembryonic antigen (CEA) targeting moieties [117, 118]. These targeted IL-2 fusion proteins were shown to have tumor localization properties and to inhibit growth of CEA expressing tumor cells in transgenic mice. These preclinical data have instigated the evaluation of CEA-targeted IL-2 in a phase I clinical trial [119, 120]. Taken together, local delivery of IL-2, as an adjuvant therapy is a very attractive and clinically relevant option, especially as adverse effects are omitted by local administration.

### Intratumoral delivery of IL-12

Although the potential of IL-12 to improve the immune stimulating capacity of DCs was discussed above; the following section will focus on its antitumor characteristics. Systemic administration of IL-12 was shown to promote antitumor immunity, counteract angiogenesis and tumor cell dissemination. This antitumor outcome is attributed to the direct inhibitory effect of IL-12 on the induction of metalloproteases, the increased lyses of endothelial cells as well as to its indirect effect on the induction of IFN-γ. The latter leads to a decreased expression of VEGF and integrin αVβ3, and an increased expression of the anti-angiogenic factors Mig-2 (CXCL9) and IP-10 (CXCL10) [121]. Moreover, IFN-γ enhances the expression of MHC molecules on DCs and of the T_H_1-polarizing factor T-bet in CD4^+^ T cells [122]. It was shown in distinct comparative studies that IL-12 is one of the most effective cytokines to eradicate experimental tumors, to prevent metastases and to induce long-term antitumor immunity [123, 124]. However, systemic administration of IL-12 is also associated with profound toxicity, including increased serum aminotransferases, stomatitis, leucopenia, thrombocytopenia and anemia [125, 126]. Different strategies have been exploited to deliver the IL-12 gene to the tumor environment, including IL-12 encoding plasmid DNA [124, 127-132] or viral vectors [133-136]. Alternative approaches have been developed to deliver the IL-12 protein such as: particles that mediate a slow IL-12 release [137, 138] or IL-12-secreting tumor-specific T cells [139, 140]. It became clear from these studies that intratumoral delivery of IL-12 can exert antitumor effects resulting in the regression of (metastatic) tumors in various models, amongst which melanoma, adenocarcinoma, renal cell, colorectal and hepatocellular carcinoma. It has been demonstrated that the antitumor effects of IL-12 within the tumor depend on CD4^+^ and CD8^+^ T cells, NK cells and/or NKT cells [141-143]. Additionally, IL-12 can functionally reprogram myeloid cells, such as MDSCs [123, 136, 144, 145]. Kerkar *et al.* showed that these cells express high levels of the functional IL-12 receptor-β2 subunit and upon IL-12 sensing they lose their suppressive activity, and can even obtain T-cell stimulating abilities [146]. These pre-clinical studies highlight that local delivery of IL-12 is an attractive approach as it can exert antitumor effects with little to no toxicity. The latter is confirmed by the clinical responses observed in patients with renal cell carcinoma, melanoma and peritoneal metastasis from ovarian cancer upon intratumoral IL-12 treatment [147-151]. Despite the encouraging results described in these studies, not all clinical trials have been successful. Triozzi *et al.* [152] were unable to demonstrate clinical benefit of intratumoral administration of canarypox-based vectors harboring IL-12 in a phase I clinical trial in metastatic melanoma. In spite of some setbacks, the relevance of IL-12 as an anticancer cytokine is undeniable and its importance in cancer immunotherapy continues to grow.

### Intratumoral delivery of TNF-α

Another potential treatment target, which gained attention thanks to the pioneering work of William Coley, is TNF-α. This cytokine is produced by various cell types upon triggering of TLR4 by LPS [153]. Different strategies have been developed to enrich the tumor environment with TNF-α, such as delivery of the TNF-α protein directly [154] or with microspheres [155, 156], delivery of the TNF-α gene using adenoviral vectors [157-159] or plasmid DNA [157]. Treatment with TNF-α was shown to (i) induce NK and T cell-mediated immunity, (ii) to reduce the Treg population and (iii) to decrease tumor cell survival [155, 157, 159, 160]. These antitumor effects could be further enhanced by combining TNF-α with other cytokines such as IL-12 [155, 160] or classical therapies, like radiation therapy [158] or chemotherapy [159], making intratumoral delivery of TNF-α an attractive adjuvant treatment.

### Intratumoral delivery of type I IFNs

Another cytokine that has been studied in the adjuvant context is IFN-α; a member of a gene family consisting of 13 members. Over half a century ago, it was discovered that this cytokine interferes with viral infections. A decade later, type I IFNs were shown to exert antitumor functions, such as tumor cell growth suppression and immune stimulation [161, 162]. Notably, IFN-α was approved by the FDA for the treatment of several cancers, including hairy cell leukemia, chronic myeloid leukemia, melanoma, renal cancer, myeloma, lymphomas and Kaposi's sarcoma. Although promising results were obtained in hematopoietic cancers, little success was achieved in solid tumors using systemic administration of IFN-α as a single agent [163]. Moreover, its systemic delivery often results in severe side effects, such as autoimmune and inflammatory symptoms and direct tissue toxicity [164]. IFN-β has also made its way to the clinic; for the treatment of multiple sclerosis, hepatitis B and to a lesser extent cancer [165-167]. Lower doses of IFN-α and IFN-β were suggested for adjuvant therapy, as this could reduce the risk of developing secondary cancers [168-170]. However, a concern with regard to the clinical use of type I IFNs is its impaired signaling. The exact mechanism is not clear yet; different factors such as immunosuppressive cytokines found in the tumor environment could be responsible. Critchley-Thorne *et al.* showed that both IFN-α and IFN-γ signaling was reduced in distinct lymphocyte subsets from patients with breast cancer, melanoma and gastrointestinal cancer [171]. However, because type I IFNs are promising therapeutic agents with a limitation on their systemic use due to toxicity, studies on the intralesional delivery of type I IFNs have been conducted. The local injection of recombinant IFNs [172], plasmid DNA encoding them [173], compounds eliciting endogenous release [62, 67] or the systemic delivery of tumor-specific antibodies coupled to type I IFNs [174, 175] were evaluated. Comparative pre-clinical studies showed the superiority of intratumoral over intravenous delivery of type I IFNs in terms of survival and toxicity [62, 176, 177].

To circumvent the toxicity issues Xuan *et al.* [175] created a fusokine composed of rituximab (anti-CD20) fused to IFN-α. This fusokine was administrated intravenously and overcame the toxicity issues linked with systemic delivery of IFN-α alone. In line with this study, Xuanming *et al.* [174] developed similar fusokines consisting of IFN-β and the antibody against Neu or EGFR and showed that therapy resistance could be overcome. This finding is of great importance as antibody resistance is a major issue for antibody-based cancer therapies. This approach focuses on the reactivation of innate and adaptive immune cells in the tumor microenvironment to overcome antibody-based resistance. Of note, both studies demonstrated that it is feasible to deliver an immune modulating molecule systemically and still achieve its local effects due to the tumor-targeting antibody that was fused to it.

The local delivery has the advantage of achieving high concentrations with minimal toxicity. This is important as many effects are exerted only at higher IFN concentrations [178, 179]. For instance, IL-6 secretion by DCs triggered with type I IFNs has been shown to be dose dependent [178]. The local increase of type I IFNs also diminishes the suppressive function of MDSCs, whilst enhancing the immune stimulating ability of DCs and the cytolytic function of NK and CD8^+^ T cells. Another advantage of type I IFNs is their ability to act on tumor cells by decreasing their proliferative rate as well as their viability [180]. In addition, tumor cells up-regulate several surface markers upon triggering of the IFN-α receptor (IFNAR1 or 2). Some of these molecules like MHC I are thought to support the antitumor immune response, whilst others are inhibitory molecules like the ligand of programmed death 1 receptor (PD-L1), which upon interaction with its receptor PD-1, expressed on effector T cells, impairs T cell responses. Given this combined effects, it is likely that type I IFNs will not be used as a monotherapy. In this regard, pre-clinical studies showed that intratumoral treatment with type I IFNs accompanied by PD-1 targeting leads to local tumor suppression [174, 181]. Moreover, combining of the intravenous antibody-IFN-β fusokines with PD-L1 blockade lead to complete eradication of tumors. In summary, the data described above suggests diverse options for the application of cytokines in the treatment of various tumors.

### Soluble receptors that capture immunosuppressive cytokines

The tumor and its microenvironment represent a potent source of anti-inflammatory and suppressive factors, which cause defects in the differentiation and maturation of immune cells, resulting in the generation of tolerogenic DCs, Tregs and MDSCs. These cells in turn can suppress the activity of effector cells in the tumor microenvironment by the secretion of soluble molecules such as IL-10, IL-35 and TGF-β [182]. Therefore, an indirect way to inhibit these negative regulators and enhance the activity of effector cells is by blocking suppressive cytokines.

### Intratumoral neutralization of TGF-β

TGF-β is the most pleiotropic and influential factor in the tumor environment as it not only affects most immune cells but also plays a key role in tissue regeneration and angiogenesis. This growth factor is known to inhibit proliferation, differentiation and maturation of T and B cells as well as to suppress the cytotoxicity of NK cells against tumor cells. Furthermore, it decreases the capacity of antigen-presenting cells to activate effector T cells and it promotes the generation and expansion of Tregs through the conversion of immature myeloid DCs. Additionally, TGF-β promotes metastasis and formation of tumor stroma [183]. Noteworthy, TGF-β has also been proposed to have a tumor-suppressing role in cancer. In a mammary tumor model, TGF-β was shown to function both as a tumor promoter and tumor suppressor [184]. In osteosarcoma, TGF-β also regulates metastatic cascades as well as normal bone remodeling and formation. Nonetheless, pre-clinical models showed that blockade of TGF-β was effective to treat and prevent bone metastases, and to increase the bone mass [185]. The therapeutic potential of this strategy was also shown in several mouse tumor models, amongst which renal cell cancer [186], melanoma [187], hepatocellular carcinoma [188], and glioma [189]. Therefore, approaches that inhibit TGF-β are under development. These include neutralizing antibodies, soluble receptors, TGF-β-binding proteins, small-molecule inhibitors, receptor kinase antagonistic drugs and antisense reagents [190, 191]. TGF-β blockade in combination with other treatments has been tested in mice for its immune stimulatory capacity. Intraperitoneal injection of a TGF-β neutralizing antibody showed enhanced cytarabine-induced apoptosis in acute myeloid leukemia cells and improved delivery and efficacy of a chemotherapeutic in two mammary carcinoma models [192, 193]. The latter was explained by enhanced intratumoral penetration of the chemotherapeutic leading to a better control of tumor growth. The efficacy of T-cell receptor gene therapy was also greatly augmented in prostate tumor bearing mice upon injection of T-cell receptor-modified CD8^+^ T cells expressing a dominant-negative TGF-β receptor II [194]. Numerous *in vitro* studies also provide rationale in favor of blocking TGF-β signaling in human tumors. For example, the introduction of dominant-negative TGF-β receptors into metastatic breast cancer cells, inhibited their epithelial-to-mesenchymal transition, motility, invasiveness, survival and metastases [195]. This was further supported by a study in which inhibition of TGF-β signaling in splenic CD8^+^ T cells isolated from F5 mice promoted the generation of CD62L^high^/CD44^high^ central memory CD8^+^ T cells. These data were confirmed in human peripheral blood mononuclear cells [196].

Importantly, the specific blockage of TGF-β, via intratumoral delivery of triple immunotherapy, consisting of anti-CD25, anti-TGF-β and anti-CTLA-4 monoclonal antibodies, in the treatment of mesothelioma, was shown to lead to the favorable therapeutic outcome [197]. Combination of these three agents led to complete tumor eradication and protection from re-challenge. The timing of intratumoral administration did not impair the outcome, as long as the three components were administered together. These data together with previous findings are in favor to target TGF-β locally.

### Intratumoral neutralization of IL-10

IL-10 has also been considered as a target to enhance antitumor immunity. It affects antigen-presenting cells directly by down-regulating MHC and co-stimulatory molecules. Additionally, it diminishes the expression of T_H_1 cytokines like IFN-γ, while it induces Tregs [198]. On that account, intravenous injection of murine IL-10 receptor blocking antibodies or oligonucleotide aptamers resulted in the inhibition of tumor growth in a colorectal cancer model [199]. When subcutaneously administered, anti-IL-10 receptor antibodies also showed a protective effect upon subsequent melanoma or anaplastic large cell lymphoma challenge. Moreover, when combined with peptide-pulsed DCs, complete protection was achieved. This was attributed to an increase of CD4^+^ granzyme^+^ T cells and a decrease in Tregs [200]. Co-administration of anti-IL-10 antibodies with LPS, induced improved immune responses. Intraperitoneal delivery of anti-IL-10 receptor antibodies in combination with BCG was effective for the treatment of bladder cancer [201]. Besides its tolerogenic properties, IL-10 also exerts anti-angiogenic effects with both tumor promoting and inhibiting results. It was concluded that IL-10 ablation promotes tumor development, growth and metastasis as tumor growth in IL-10 knock out mice was associated with an increased level of MDSCs and Tregs in both the tumor environment and in the tumor-draining lymph nodes [202]. Therefore, caution should be taken with anti-IL-10 treatment during pre-clinical evaluation.

Of note, IL-19, a member of the IL-10 family, which also contributes to a range of diseases, was recently linked to the progression of cancer [203]. In breast cancer for example, this cytokine is correlated with an increase of mitotic figures, advanced tumor stage, higher metastasis, and poor survival. IL-19 has an autocrine effect on breast cancer cells by directly promoting proliferation and migration of tumor cells while indirectly providing a microenvironment for tumor progression. This suggests a prognostic value of IL-19 and a possible therapeutic potential of its antagonists.

### Intratumoral neutralization of IL-35

Apart from TGF-β and IL-10, expression of the Treg-associated cytokine IL-35 has been demonstrated in the tumor environment. It appears to promote tumor growth by enhancing myeloid cell accumulation and angiogenesis, hereby weakening spontaneous CTL responses [204]. Although it has been shown that IL-35 is mainly produced by Tregs, gene expression analysis has revealed that IL-35 has likely a wider distribution, including expression by human cancer cells such as melanoma. The presence of IL-35 seemed to correlate with the severity of the disease and the clinical stage of the tumor in colorectal cancer [205]. Interestingly, it was shown for human prostate cancer that Tregs mediate their effects *via* CTLA-4 and IL-35 while IL-10 or TGF-β blockade does not abrogate their suppressive activity [206]. It has been also observed that IL-35 over expression increases apoptosis sensitivity and suppresses cell growth in human cancer cells [207]. Likewise as with IL-10, caution is needed when evaluating anti-IL-35 treatments for cancer.

In conclusion, several immunosuppressive cytokines are present in the tumor microenvironment; in depth understanding of their pro- and antitumorigenic roles, which depend on the species, tumor type and tissue as well as its inflammatory status is crucial for the success of therapy involving these molecules.

### Fusokines: combining the best of two worlds

Intratumoral delivery of cytokines or blockade of immunosuppressive cytokines showed promising immunological and clinical benefit, however not in all patients. Combination of immune modulators could help to achieve the required therapeutic potency, especially given that the resistance to a certain immune modulator can occur. Since we theoretically could combine an infinite amount of cytokines and blocking agents, an important question that arises is: ‘Which combination should be used for which cancer type’? Screening of the patients for the presence of inhibitory mechanisms, tumor cytokines or genes encoding them would serve for the proper design of the treatment.

Penafuerte *et al.* developed a fusokine by combining IL-2 and the ectodomain of the TGF-β receptor II. When delivered intratumorally, this fusokine, named FIST, acts as a “sword and shield” strategy by trapping locally produced TGF-β and stimulating lymphocytes with IL-2. FIST was shown to recruit NK cells, NKT cells, CD8^+^ T cells and B cells to the tumor. In addition, the lymphocytes were shown to secrete higher levels of IFN-γ upon FIST stimulation.

**Figure 3:**
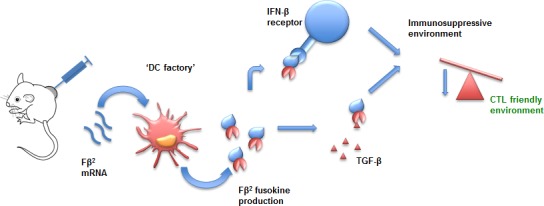
mRNA encoding for Fβ^2^ as a tool for intratumoral immunomodulation mRNA encoding Fβ^2^, a fusokine consisting of IFN-β fused to the ectodomain of the TGF-β receptor II, is taken up by tumor-residing DCs. These DCs serve as factories to produce therapeutic amounts of the fusokine. This fusokine creates a CTL-friendly environment and as such contributes to antitumor immunity. Abbreviations: CTL (Cytotoxic T Lymphocytes); DC (Dendritic Cell); IFN-β (Interferon-β); TGF-β (Transforming-Growth Factor-β).

Inspired by these findings as well as the observation that IFN-β could be combined with a TGF-β signaling agonist [208], we generated mRNA encoding for a fusokine named Fβ^2^, consisting of IFN-β and the ectodomain of the TGF-β receptor II (Fig. 3) [181]. The Fβ^2^ fusokine reduced the suppressive capacity of MDSCs and increased the stimulatory capacity of DCs. Moreover, CD8^+^ CTLs showed enhanced tumor cell recognition upon exposure of tumor cells to the fusokine. In tumor bearing mice, depletion of CD8^+^ T cells abrogated the antitumor effect. This approach offers potent immune stimulation devoid of TGF-β mediated suppressive effects. Similarly to Xuanming *et al.* [174] we [181] obtained improved therapeutic responses via specific targeting of the type I IFN induced PD-L1 up-regulation.

**Figure 4:**
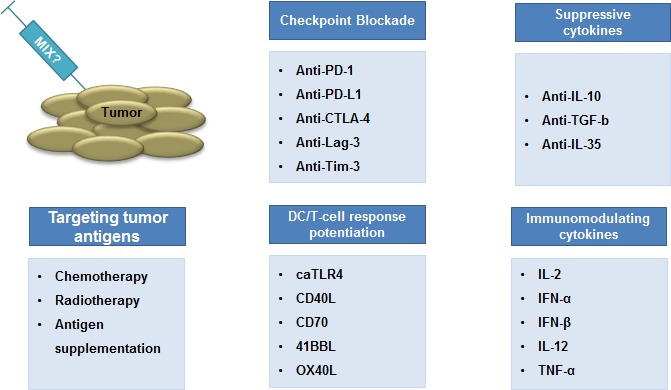
Combination therapy: the way forward The combination of cytotoxic pharmaceutics is a standard strategy in clinical oncology to increase clinical responses. A similar paradigm has emerged in cancer immunotherapy. It is believed that strategies that aim at inducing tumor-specific T cells should be combined with strategies that counteract mechanisms devised by the tumor and its environment to down-regulate T cell-mediated tumor cell rejection. We contend that smart combinations of the strategies discussed in this review, in particular immunization and immunomodulation, are key to the eradication of some of the currently difficult to treat tumors.

These studies highlight the complexity of tumors and their environment and show that immunotherapeutics can have diverse effects, resulting in a delicate balance between immune stimulation and immune inhibition. Tipping this balance to immune stimulation in order to have effective antitumor responses will most likely be obtained through combination of multiple therapies (Fig. 4). In this regard, immune checkpoints have increasingly gained attention, as these are believed to majorly impact on immune-mediated tumor rejection.

### Immune checkpoint blockade

The ability of cancer cells to suppress antitumor T-cell activity in their microenvironment was pinpointed by Hanahan and Weinberg as one of the hallmarks of cancer progression [209]. Multiple inhibitory ligands and receptors expressed by tumor cells, tumor-infiltrating myeloid cells and lymphocytes can account for this anticancer activity. T-cell activation requires antigen recognition by the T-cell receptor as well as complementary signals, coming from co-stimulatory molecules and cytokines. The opposite, T-cell ignorance, is mediated by inhibitory checkpoints that hamper proper activation. These inhibitory checkpoints are known to down-regulate effective antitumor immunity. Therefore immune checkpoints have gained increasing attention as novel therapeutic targets. In 2013, the editors of ‘Science’ proclaimed cancer immunotherapy, more specifically the blockade of inhibitory immune checkpoints, as ‘Breakthrough of the year’ [210]. The relevance of targeting immune checkpoint molecules is highlighted by the numerous reviews that have recently been published [211-219].

Antibodies against CTLA-4 and members of the PD-1/PD-L1 pathway have been extensively and successfully studied in both the pre-clinical and clinical setting. However, there are many other molecules that are able to down-regulate T-cell functions, amongst which T-cell immunoglobulin and mucin domain-containing protein-3 (Tim-3) and Lymphocyte activation gene-3 (Lag-3). Therefore, it is not surprising that monoclonal antibodies against Tim-3 and Lag-3 are also under development [220-222].

Of the monoclonal antibodies that were generated only those directed against CTLA-4 have been studied for local delivery. CTLA-4 is a regulator of early stage T-cell activation in response to antigen; it puts a brake on T cells, preventing them from launching uncontrolled immune attacks [223]. CTLA-4 is a homolog of CD28 and plays an important role in the development of peripheral tolerance to self-proteins [224, 225]. The main ligands for CTLA-4 are B7-1 (CD80) and B7-2 (CD86), which transmit an inhibitory signal to CTLA-4 expressing T cells. Diverse anti-CTLA-4 monoclonal antibodies are developed of which ipilimumab, a human IgG1 anti-CTLA-4 antibody, and tremelimumab, an IgG2 anti-CTLA-4 antibody, are best known. Currently, ipilimumab is broadly used in clinical trials and already demonstrated the potential to improve the overall survival of cancer patients, either as a single agent or in combination with other therapies [226-231]. Treatment with tremelimumab was shown to enhance the diversity of the T-cell receptor repertoire and increase the total T-cell number, without an expansion of specific clones [232]. To have a better insight in the clinical benefit for anti-CTLA-4 treatment, the tumor genetic landscape was scrutinized. Interestingly, it was shown that patients, which tumors possessed certain neoepitopes displayed a prolonged benefit from the anti-CTLA-4 blockade [233, 234]. The effects of the therapy are not only related to the effector T-cell function but also to the interference with Tregs. In this regard, it has recently been shown in mice that treatment with anti-CTLA-4 monoclonal antibodies might deplete Tregs in the tumor microenvironment [235]. However, immune-related auto-reactivity and toxicity have been reported and can be explained by the fact that CTLA-4 plays a critical role as a negative regulator of T-cell activation [214]. Moreover, CTLA-4 knock out mice develop autoimmune disease which is lethal at the age of 3 to 5 weeks. Nevertheless, Fransen *et al.* described efficient systemic activation of tumor-specific T cells by the peri-tumoral administration of CTLA-4-blocking antibodies in the lipid-based adjuvant montanide [213, 236]. Using a slow-release formulation they could drastically reduce side effects and decrease the risk of autoimmune reactions without losing the systemic effect. Similarly, Marabelle *et al*. [101] co-injected intratumorally ODNs, anti-CTLA-4 and anti-OX40 antibodies, and achieved superior effects to those obtained by the systemic administration of a 100-fold higher dose of anti-CTLA-4 antibodies. In this approach, tumor-residing Tregs were depleted, which boosted the immune responses. Both of these pre-clinical studies highlighted the value of the intratumoral approach and showed that local delivery of anti-CTLA-4 exerts similar antitumor responses to its systemic administration.

## GENERAL CONCLUSIONS

A great effort was made to develop treatment modalities that directly target the tumor. As outlined throughout the review, intratumoral administration of immunotherapies holds several advantages and only a few disadvantages when compared to their systemic delivery. The pro's and con's of using the tumor as a site of immunotherapy are summarized in Table 1.

**Table 1:** Advantages and disadvantages of using the tumor as a site of immunotherapy

Advantages	Disadvantages
No need to identify antigens or HLA for immunization purposes.	Tumors are not always accessible.
Site of manipulation is the site of action for the effector cells.	
Ability to manipulate the tumor environment.	Intratumoral therapy has to proceed surgical removal of the tumor, which entails a delay of this classical therapy.
Local therapy can result in systemic responses.	
Local therapy is contended to reduce therapy-related toxicity.	

Modulation of the tumor microenvironment appears as an elegant way to generate therapeutic responses. However supplementation of a single factor, except for some cases, is often not resulting in beneficial outcome. Therefore, it is essential to combine different stimulatory factors with proper alleviation of inhibitory mechanisms. It still needs to be addressed whether a golden standard mix, which suits most of the tumors or rather tumor-specific mix would offer greater therapeutic benefit. In this regard, clinical data from ongoing and future studies will provide valuable information.

The evolution of cancer research clearly showed that this disease is highly potent in countering drugs aiming at destroying it. Therefore, a certain immune boost is needed to overcome the development of escape mechanisms and to generate strong memory responses. In (Fig. 4) we propose the components of an ideal mix that should consist of 1) proper DC maturation and T-cell stimulating signals, 2) blockade of inhibitory immune checkpoints and 3) neutralizers of immunosuppressive cytokines. We believe that the combinatorial therapies are the future of cancer treatment and that they will bring a clear benefit for the patients in the coming years.
